# Development and Characterization of Sr-Containing Glass-Ceramic Composites Based on Biogenic Hydroxyapatite

**DOI:** 10.1186/s11671-018-2550-1

**Published:** 2018-05-16

**Authors:** Oleksii Kuda, Nataliia Pinchuk, Oleksandr Bykov, Tamara Tomila, Olena Olifan, Maryna Golovkova

**Affiliations:** Frantsevich Institute for Problems of Materials Science of NAS of Ukraine, 3, Krzhyzhanovsky Str, Kyiv, 03680 Ukraine

**Keywords:** Hydroxyapatite, Calcium phosphate, Glass, Composite, Strontium, XRD, Porosity

## Abstract

Composite materials based on hydroxyapatite are widely used for bone tissue engineering. There is evidence of a positive effect of the presence of strontium in osteoplastic materials in the case of a Ca/Sr certain ratio. To examine the effect of the addition of Sr^2+^, a study was made by introducing it into the material composition based on biogenic hydroxyapatite and sodium borosilicate glass (50/50% wt.). The strontium was introduced into the composition in an amount of 1% wt. Composite materials were obtained at final sintering temperatures of 780 °C and a sintering time of 1 h. The effect of additions of glass phase and strontium affect changes in the crystal lattice of biogenic hydroxyapatite was investigated with the help of X-ray phase analysis, IR spectroscopy. Also the behavior of composites in vitro in physiological solution was studied.

## Background

Hydroxyapatite (HA) and other bioactive calcium phosphate materials, including bioactive glass, can be used in tissue engineering to replace bone tissue. Natural biogenic hydroxyapatite (BHA) is nano-structural material, nano-, and microstructure of which is analogous to mineral component of bone tissue.

Earlier production results were received, and various types of composites on the base of biogenic and synthetic hydroxyapatite and glass phase were studied [1–5]. It is known that, in the chemical composition of natural biogenic hydroxyapatite (BHA), there are small amounts of other non-organic compounds such as Ca_4_O(PO_4_)_2_, NaCaPO_4_, Ca_3_(PO_4_)_2_, CaO, and MgO, as well as traces of Al^3+^, Fe^3+^, Mg^2+^, Sr^2+^, K^+^, Si^4+^, Na^+^, Cu^2+^, Zn^2+^, and CO_3_^2−^[6–9]. Therefore, composites of BHA/glass with the use of various bioactive additives that can contribute to the processes of osteogenesis and formation of new bone tissue were also studied and obtained. These composites were alloyed by ion additives Fe^2+^/Fe^3+^ and Cu^2+^ [10, 11], Ce^2+^ [12], Si^4+^ [13], La^3+^ [14], and other.

A modern problem in the field of diseases of bone pathology is the treatment of osteoporosis that is the rarefaction of the density of the patient’s bone tissue as a result of hormonal or other causes. That is why it is quite relevant to develop new bioactive materials that can stimulate the formation of new cells of bone tissue and improve its structure. From this point of view, the introduction of strontium ions into the composition of calcium phosphate materials can improve their bioactive properties [15–17].

Development of instruments used for bone tissue regeneration in vivo is focused on cellular models and processes of differentiation.

The authors [15, 18] point at the role of Sr^2+^in maintaining bone regeneration during the process of skeletal restoration as a whole, and more specifically, in application of cellular therapy. For example, strontium ranelatere presents itself an interesting compound that stimulates bone formation and inhibits bone resorption. In the estimation of effect of the wide range of strontium (Sr^2+^) concentration in vitro*,* the authors established that Sr^2+^ promoted a proliferation of PA20-h5 cells as well as HA formation in the course of osteoinduction in vitro. Data mentioned above point to the role of Sr^2+^ in maintaining bone regeneration during the process of skeletal restoration as a whole, and more specifically, in application of cellular therapy.

The authors of this work [17] have received stable suspensions of nanopowders of strontium-containing hydroxyapatite (Sr-HA) by method of precipitation from aqueous solution (strontium content in the amount from 0 to 100 mol%). Studies of materials obtained point to the complete solubility of strontium in hydroxyapatite lattice in isomorphic replacement of Ca^2+^ for Sr^2+^. Addition of strontium is responsible for the increase of ratio *c/a* in the triclinic elementary cell. A significant change in the form and size of nanopowders is observed, in which connection a prevailing growth is in the direction along *c* axis manifested in case of higher strontium content.

Data of suspensions biocompatibility studies, from the point of view of viability of cells, apoptosis, proliferation, and morphology with the use of cell line of osteosarcoma SAOS-2 point to the increased proliferation of cells for HA nanoparticles containing greater number of Sr^2 +^, in so doing, it has, practically, no effect on morphology of cells.

In the work [19], multifunctional porous granules were developed as fillers of bone tissue and carriers of medicinal agents. Powders of strontium and magnesium-replaced material HA/TCP with compositions close to the mineral part of human bone [(Ca + Sr + Mg)/*P* = 1,62], have been obtained by precipitation, then subjected to heat treatment and deagglomeration. Drying and sintering permitted to receive porous granules that were saturated with antibiotic (levofloxacin) solution, frozen, and then lyophilized. The estimation of medicinal agent release and osteocompatibility of granules has shown that Sr-alloyed granules have demonstrated the highest levels of proliferation and effectiveness in osteoblastic maturation.

There are some data about the influence of strontium additive on the behavior of bioactive glass 1393 nBG particles in vitro, from the point of view of physic-chemical reactions occurring on their surface in SBF [20]. In comparison with non-alloyed glass 1393 nBG, it was found out that apatite formation is slowed down in Sr introduction that, probably, is the result of inhibiting action of Sr ions on HA crystallization. In addition, it was revealed that crystallinity of precipitated calcium phosphate was lower in the case with Sr-alloyed glass 1393 nBG compared to a pure standard material 1393 nBG. The authors of the abovementioned work also point out that the ability to form HA bioactive glasses gives the notion about their surface reactivity that has relation to the application of the nano-scaled particles of biologically active glass in bone regeneration.

Studies carried out in vivo on rats by the authors of work [21] have shown positive impact of strontium-containing macroporous bioactive glass (Sr-MBG) on the process of bone formation as well as on the decrease of bone resorption in rats with osteoporosis (osteoporotic rats). Investigation has demonstrated that the use of (Sr-MBG) leads to the increase of bone formation (46.67%) in comparison with non-alloyed glass MBG (39.33%) and control group (17.50%).

The aim of the work is preparation and investigation of bioactive materials based on biogenic hydroxyapatite/glass composite with SrО-additive for tissue engineering.

## Methods/Experimental

### Sample Preparation

Bioactive glass-ceramic composites based on nanostructured biogenic hydroxyapatite (BHA) with addition of sodium borosilicate glass (mass%: 46 SiO_2_; 28 B_2_O_3_; 26 Na_2_O) were prepared as described in [11]. To obtain Sr-containing glass-ceramic composites samples were used powders of bioactive glass-ceramic composites based on nanostructured biogenic hydroxyapatite (BHA) and sodium borosilicate glass with the relation 50/50% wt and sintered at the temperature 1100 °C (start sintering), then were crushed. Obtained powders of the composites were mixed with powder of strontium oxide (1 wt.%). The samples of BHA/glass and BHA/glass-Sr composites of 2.5 g and 11 mm diameter were formed and sintered at the temperatures 780 °C.

### Characterization Methods

The phase composition and structure of the obtained samples were studied by X-ray diffraction, IR spectroscopy, and SEM.

### X-ray Diffraction

For X-ray analysis, the X-ray diffractometer DRON-3M was used equipped with additional computer system of scanning, X-ray tube with copper anode and nickel filter. Thus, diffraction images were obtained with the help of Cu–Kα radiation with a medium wavelength λ = 1,54178 Ǻ3.

### IR-Spectroscopy

In addition, the materials were studied by infrared (IR) spectroscopy using a spectrophotometer FSM 1202 (TOV Infraspectr, Russia) in the wavenumber range 4000–400 cm^− 1^.

### Structure

The structure of the composites was studied by scanning electron microscopy (SEM) using a REM-106I (VAT SELMI, Ukraine).

### Porosity

The composite samples were examined for the apparent density and total and open porosity (*Θ*_*t*_ and *Θ*_op_). The total porosity of samples (%) was calculated using the following formula:

$$ {\Theta}_{\mathrm{t}}=\left(1-{\uprho}_{\mathrm{ap}}/{\uprho}_{\mathrm{pykn}}\right)\cdotp 100, $$

where *ρ*_ap_ is the apparent density, g/cm^3^;

ρ_pykn_ is the pycnometric density of compact material, g/cm^3^.

For BHA *ρ*_pykn_ = 3.00 g/cm^3^.

In order to determine the open porosity, a sample was weighted and saturated with ethylene in vacuum. The saturated samples were weighted in water and in air. The open porosity of samples (%) was calculated by the formula:

$$ {\Theta}_{\mathrm{op}}=\left({\mathrm{m}}_1-\mathrm{m}\right)\cdotp {\uprho}_{\mathrm{w}/}\left({\mathrm{m}}_1-{\mathrm{m}}_2\right)\cdotp {\uprho}_{\mathrm{liq}}, $$

where *m* is the sample weight in air, g;

*m*_*1*_ is the saturated sample weight in air, g;

*m*_*2*_ is the saturated sample weight in water, g;

*ρ*_*w*_ is the density of water, g/cm^3^;

*ρ*_liq_ is the density of the saturating liquid (ethylene), g/cm^3^.

The closed porosity of samples was calculated by the following formula:

$$ {\Theta}_{\mathrm{cal}}={\Theta}_{\mathrm{t}}-{\Theta}_{\mathrm{op}} $$

### In Vitro Bioactivity Testing

Investigation of solubility in vitro of porous samples was carried out in an isotonic saline solution (0.9% NaCl) at a solid/liquid ratio of 1:30 after 2, 5, and 7 days exposition in a thermostat at 36.5 ± 0.5 °C followed by determination of mass loss on an analytic balance “OHAUS Pioneer PA214C” (OHAUS Corporation, China) with an accuracy of 0.0001 g.

## Results and Discussion

**Fig. 1:**
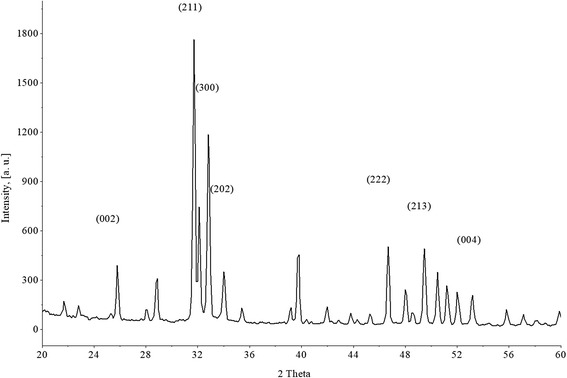
XRD patterns for initial BHA

**Fig. 2:**
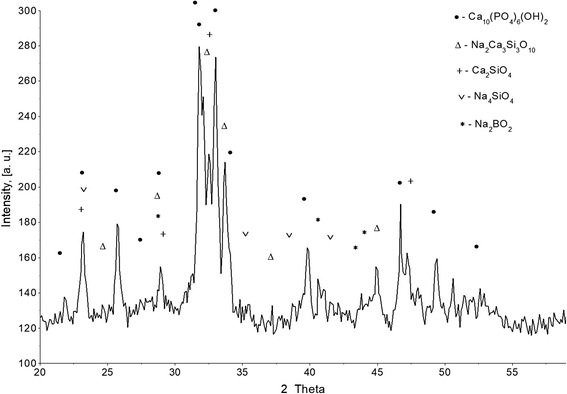
XRD pattern of BHA-glass composite with the notation of the most intense peaks of the main phases

**Fig. 3:**
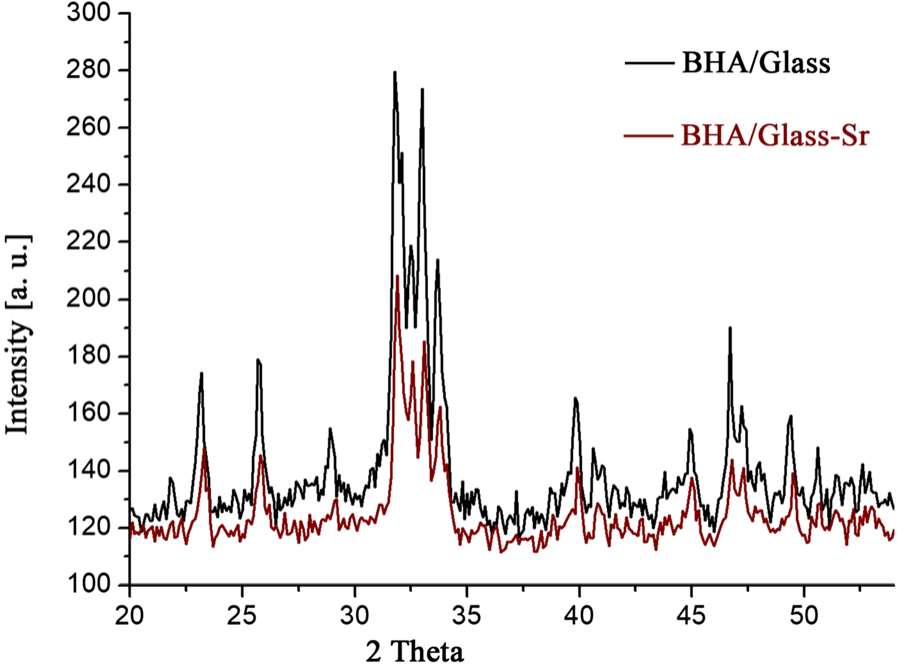
XRD patterns for both composites BHA/glass and BHA/glass-Sr.

**Fig. 4:**
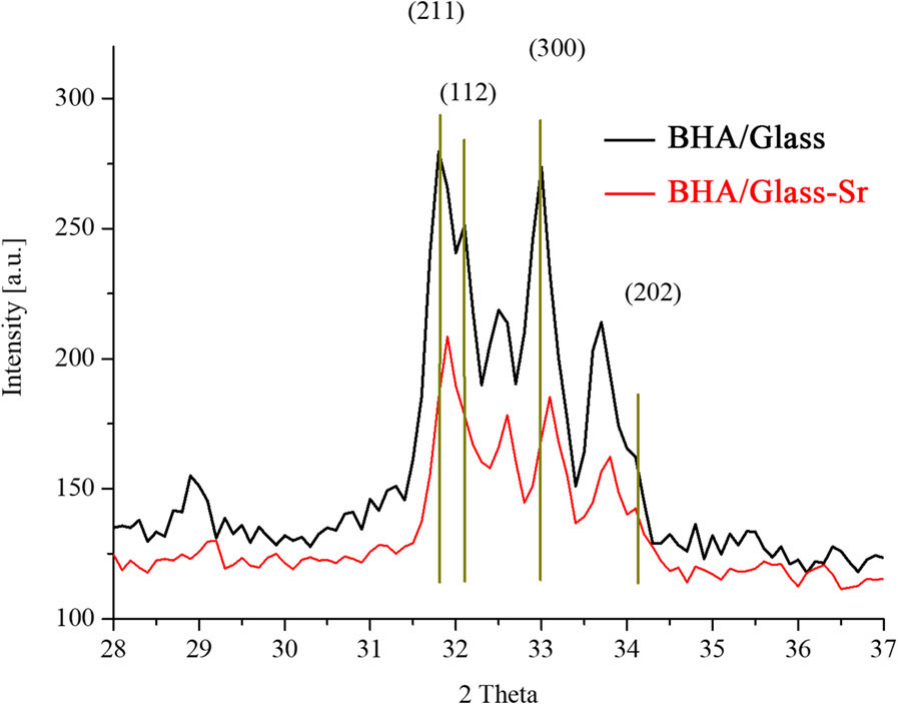
XRD patterns for both composites BHA/glass and BHA/glass-Sr for planes (211), (112), (300), and (202)

The results of the study of the phase composition of initial BHA and prepared composites are shown in Figs. 1, 2, 3, and 4. It was established that HA maintains its phase composition in BHA/glass composites.

Initial powder BGA demonstrates a diffraction image corresponding to PDF file 72-1243 (Tables JCPDS) (Fig. 1). Indicated standard diffraction image belongs to crystals with hexagonal primitive crystal lattice having periods of *а* = 9432 and *с* = 6881 Å. Calculation of periods is performed with the use of peaks (002), (211), (300), (222), and (213), lying in the range of 2θ = 24^°^–52^°^. Accordingly, the volume of elementary crystal cell of such crystals is equal to 530,496 Ǻ^3^, that insignificantly differs from that indicated in the standard file value – 530.14 Ǻ^3^.

At the expense of presence of amorphous phase in the composite BHA/glass, a process of liquid-phase sintering occurs. As the result of active interaction of components of glass phase and ВНА, a formation of new crystal phases takes place. By the results of XPA were determined basic crystal phases of BHA/glass composite (Fig. 2). It was established that obtained composites are heterogeneous material. The following phases are presented with the most intensive peaks: Ca_10_(PO_4_)_6_(OH)_2_, Na_2_Ca_3_Si_3_O_10_, Ca_2_SiO_4_, Na_4_SiO_4_, Na_2_BO_2_.

The volume of crystal cell depends on its perfection, i.e., appropriate filling with ions forming a part of hydroxyapatite. Structural features of elementary HA cell are studied sufficiently deep [9, 22, 23]. Using existing data, we can state that a plane (004) has in its composition atoms of phosphorus, oxygen, and calcium, while a plane (211) is formed with atoms of oxygen and (202)– with ions Са_II_^2+^. The main amount of oxygen is in the composition of tetrahedrons РО_4_^3−^. Since the relative intensity depends on atomic factor of dissipation (other factors are identical), a relative intensity of peak (202)–*I*_(202)_/*I*_(211)_ can indirectly testify to the changes in the number of vacancies in ions Са^2+^ or their absence. It should be noted that atomic scattering factor for Cu-***K***_α_ radiation at sinθ/λ=0.5 for Са^2+^ ions is equal to 8.1, and for Sr^2+^ ions – 19.6 [24]. Replacement of ions Са^2+^ for Sr^2+^ ions increases periods *а* and *с* of crystal lattice in hydroxyapatite [25]. So, if the above replacement leads to a formula of solid solution Ca_10−*x*_Sr_*x*_ (PO_4_)_6_(OH)_2_, where *х* = 1, then a volume of the elementary crystal cell exceeds the value ~ 540 Ǻ^3^.

Introduction of strontium oxide in the amount of 1% by mass into the composition of BHA/glass does not substantially change a phase composition of composites; this can be seen in comparison of diffraction images, located in one diagram without any change of intensity (Figs. 3 and 4).

However, there are differences in the position of centers of peaks BHA/glass and BHA/glass-Sr, as well as the decrease in the intensity of peaks and their shift to the side of large angles in BHA/glass-Sr (Fig. 4). Probably, that strontium introduction into BHA/glass leads to the decrease of the number of crystal phases compared to a non-alloyed composite, as well as contributes to the formation of crystalline structure of composite at the expense of transition of crystal phases to amorphous (Fig. 3). In Fig. 4, main peaks of ВНА are given that are expanded in the angular range 2θ and designated with vertical lines.

The internal volume of the crystal was calculated accordingly to the composition of flawless hydroxyapatite Ca_10_(PO_4_)_6_(OH)_2_, corresponding to PDF file 72-1243 (Tables JCPDS). Carried out calculation of dimensions of crystal lattices by the results of XPA indicates the changes in the periods *а* and *с*, and the volume of lattice as well (Table 1). It is evident that in case of intensive interaction with components of composite, strontium causes a degradation of crystalline structure, leads to the decrease of inter plane distances and, accordingly, decrease of volume of BHA elementary crystal cell. Data given in the authors’ work [25] point to the increase of basic parameters of lattice (periods of *а* and *с*, volume of crystal lattice) НА in sol-gel replacement of Sr in НА. However, as it was stated above, material we have studied is based on biogenic hydroxyapatite and contains 50% by mass glass phase. Probably, in the interaction of ВНА with glass phase occurs the formation of new crystal phases (Fig. 2), which, theoretically, can lead to the change of ratio Са/Р and exerts influence on the formation of vacancies Са^2+^. These changes can lead to the change of periods *а*, *с*, and volume of the elementary lattice (Table 1). Additional introduction of strontium into BHA/glass composite increases the influence on parameters of HA elementary lattice.

**Table 1:** Parameters of HA crystal structure in BHA/glass composites

Parameter	Composite material		
	Initial BHA	BHA/glass	BHA/glass-Sr
*а*, Ǻ	9.432	9.410	9.391
*с*, Ǻ	6.885	6.911	6.903
*V*, Ǻ^3^	530.496	529.976	527.205

As it is known [26], hydroxyapatite spectrum is characterized with two intensive groups of bands about 1040 and 570 cm^− 1^.

**Fig. 5:**
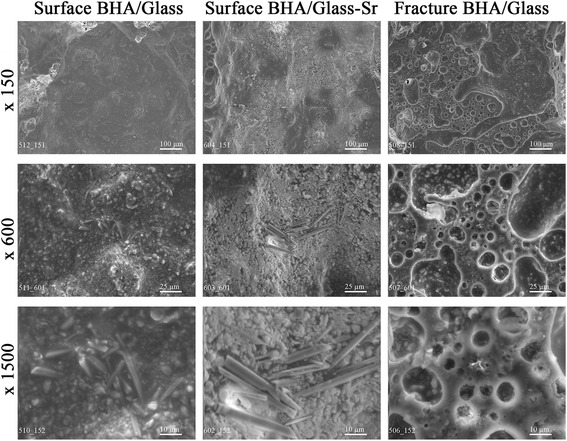
IR-spectra for both composites BHA/glass and BHA/glass-Sr.

Analysis of IR-spectra of composites absorption on the base of BHA and sodiumborosilicate glass shows that for such a system, a superposition of spectra of BHA and sodium borosilicate glass is typical (Fig. 5). A complete assignment of IR frequencies of composites on the base of HA and sodium borosilicate glass, we have described in the work [27]. Introduction of 1% strontium into such composition leads to a significant expansion of all bands of absorption of studied IR spectrum and change of shape, frequencies of absorption bands in the range of ν ~ 1050–700 cm^− 1^ and shift of band from ν ~ 1045 cm^− 1^to a long wavelength side compared to the initial BHA (Fig. 5). Such changes can be associated with the influence of strontium addition into the studied composition system. However, super-positions of areas of the absorption bands manifestation in the studied composition, makes the unequivocal interpretation difficult. It should be noted that presence of admixed atoms and other defects substantially influence on the oscillating IR-spectra. In the investigated specimens, presence of СО_2_ from the atmosphere and a small amount of residual organic products of reaction (1900–2000 cm^−1^) are observed.

**Fig. 6:**
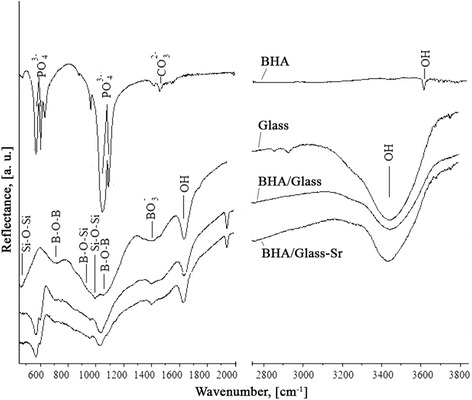
Porosity structure of both composites BHA/glass and BHA/glass-Sr.

The results of investigation for the total porosity and its open and closed fractions are presented in Fig. 6. It is shown that total porosity of composites BHА/glass-Sr practically by 2 times exceeds that of composites BHA/glass and can reach 61%. Such high porosity enables the composite to become a structural analogue of bone tissue, total porosity for which is in the range of 55–70% [7]. Here, the share of open porosity for composites doped with strontium is also higher, than for undoped composites. In introduction of strontium, open porosity increases from 6 to 10% (Fig. 6).

In our case, strontium was introduced into the prepared composite material that, probably, involved a change in the composition of glass phase with weakening in the structure of silicon-oxygen framework. As the result of the above processes, it decreases a viscosity that is the basic factor determining the diffusion processes in sintering [28] and causes foaming of crystalline glass composite. And as the result, probably, it leads to the increase of interaction on the surface of ВНА/glass, i.e., ion exchange between ВНА and glass phase is increased. Probably, it leads to a partial replacement of calcium ions for strontium ions in the structure of ВНА, with subsequent transition of calcium ions into glass phase with formation of new crystal phases that leads to the subsequent changes not only in crystal lattice, but in the structure of the whole composite.

**Fig. 7:**
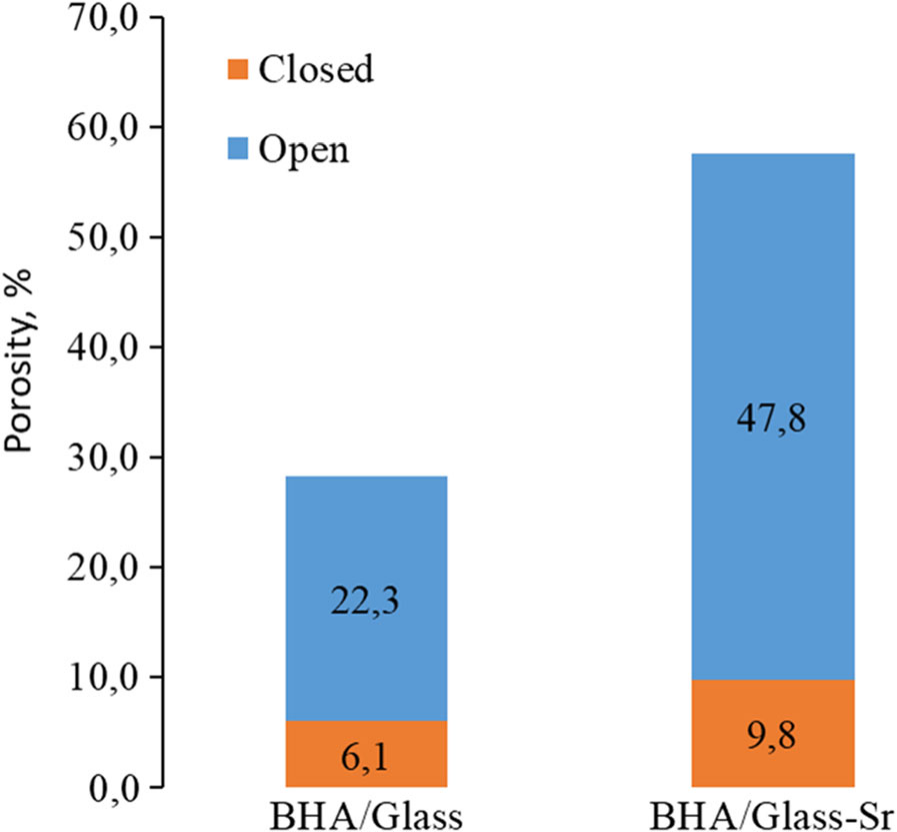
Microstructure of both composites BHA/glass and BHA/glass-Sr.

Figure 7 represents the microstructure of surface and fracture of composites studied. From microphotographs given, we can see that the structure of BHA/glass and BHA/glass-Sr can be characterized by matrix structure that is formed at the expense of some features of liquid phase sintering of material. Here, sodium borosilicate glass phase creates a “framework”, in which there are crystal particles of hydroxyapatite. In so doing, a pore size composes a wide range from 1 to 600 μ. Availability of pores of different sizes in the material of BHA/glass and BHA/glass-Sr is associated, in our opinion, with different nature of pore formation: pores with the size 100–600 μ are formed at the expense of inter-granular free spaces; pores with the size less than 100 μ—at the expense of glass mass foaming in final sintering [10]. Agglomerates in the structure of composite are formed from particles with the size > 0.2 μ. Here, on the surface, we clearly see agglomerate formations of crystals with distinct shape and sizes from 1 to 4 μ in transverse section, and which reach 30 μ in length for BHA/glass-Sr. In the process, formation of these agglomerates of crystals is observed exclusively on the surface of composite specimens.

As a whole, it is evident that the microstructure of the internal part of samples is polyporous, in other words, it is characterized by the presence of multi-sized pores, which is promising in view of the achievement of high resorption properties of bioceramics under direct contact with human organism media.

**Fig. 8:**
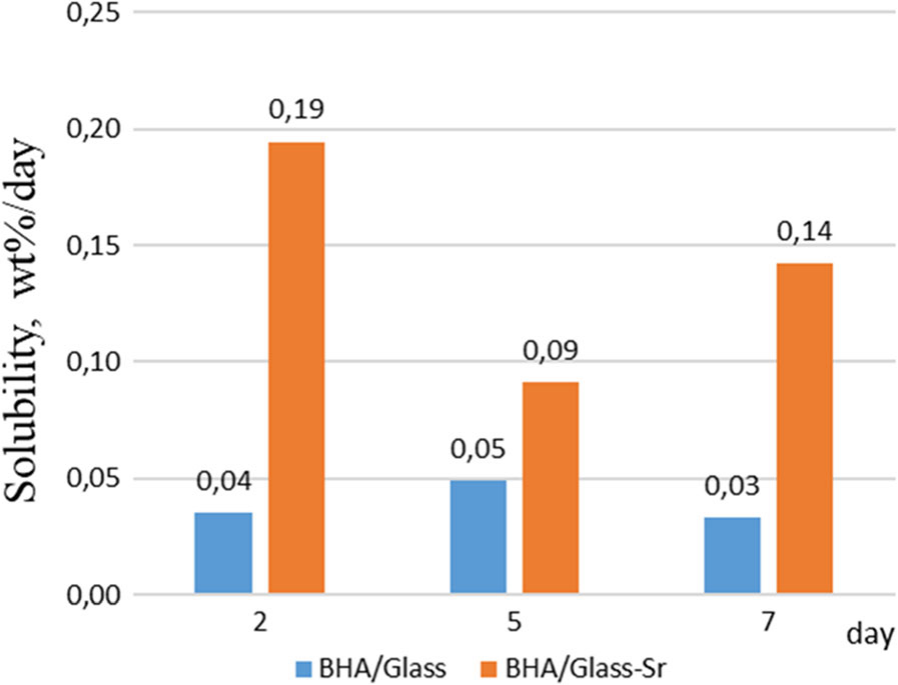
Dissolution rate in saline of both composites BHA/glass and BHA/glass-Sr (2, 5, 7 days)

Investigation in vitro after 2, 5 and 7 days of saline exposure of all composites studied revealed that the solubility of BHA/glass-Sr composites are higher than BHA/glass composites (Fig. 8). As it is seen from Fig. 8, dissolution rate of composite specimens of BHA/glass-Sr in physiologic solution exceeds that for BHA/glass composites by 2–4 times depending on the time of being in physiologic solution. The highest rate of dissolution is registered for BHA/glass-Sr composites after 2 days of staying in physiologic solution and constitutes 0.19% mass/day. A nature of dynamics of dissolution rate for composites differs for both types of composites.

Since the content of glass phase in both types of composites is practically identical, therefore, the influence of its quantity on the dissolution will be also analogous. Thus, the results obtained may be caused by significantly higher porosity of BHA/glass-Sr composites, as well as by greater share of open porosity compared to BHA/glass composites.

Obtained composites can be applied in medicine to replace some defective areas of patients’ bone tissue. Availability of the wide range of pore sizes in the obtained materials enables to use them in various regions of human skeleton.

## Conclusions

New, strontium-doped composite materials are obtained on the base of biogenic hydroxyapatite and glass phase. XPA showed that obtained composites are heterogeneous material. Content of glass phase and strontium as alloyed additive in the composite material of BHA/glass has influence on the change of parameters of BHA elementary cell, namely, leads to the decrease of crystal lattice of hydroxyapatite. It was also established that BHA/glass-Sr composite possesses a higher porosity as well as higher rate of dissolution in physiologic solution that makes the composites obtained as perspective materials to be used in medicine for replacement of defective areas of patients’ bone tissue.
